# Fermented Mangosteen (*Garcinia mangostana* L.) Supplementation in the Prevention of HPV-Induced Cervical Cancer: From Mechanisms to Clinical Outcomes

**DOI:** 10.3390/cancers14194707

**Published:** 2022-09-27

**Authors:** Zaira Kharaeva, Pavel Trakhtman, Ilya Trakhtman, Chiara De Luca, Wolfgang Mayer, Jessie Chung, Galina Ibragimova, Liudmila Korkina

**Affiliations:** 1 Microbiology, Immunology, and Virology Department, Berbekov’s Kabardino-Balkar State Medical University, Chernishevskiy Str. 176, 360000 Nalchik, Russia; 2 Blood Bank, Dmitry Rogachev National Medical Research Center of Pediatric Hematology, Oncology and Immunology, Samora Mashela Str. 1, 117988 Moscow, Russia; 3 R&D Department, Swiss Dekotra GmbH, Badenerstrasse 549, CH-8048 Zurich, Switzerland; 4 R&D Department, Medena AG, Industriestrasse 16, CH-8910 Affoltern-am-Albis, Switzerland; 5 Natural Health Farm Ltd., 39 Jalan Pengacara U1/48, Temasya Industrial Park, Shah Alam 40150, Selangor, Malaysia; 6 Centre for Innovative Biotechnological Investigations Nanolab (CIBI-NANOLAB), Vernadskiy Pr. 97, 117437 Moscow, Russia

**Keywords:** apoptosis, cervical carcinogenesis, fermented mangosteen, HPV, IL-2, myeloperoxidase, nitric oxide metabolites, sFAS, TRAIL, TNF-alpha

## Abstract

**Simple Summary:**

Human papillomavirus (HPV) is connected with virtually all cases of cervical cancer. The viral infection-associated chronic inflammation, oxidative stress, and alterations in apoptosis have been considered as leading risk factors for carcinogenesis in humans. In an observational clinical study, we identified oxidative markers and the cervical/circulating ligands of TNF-alpha-induced apoptosis involved in HPV-associated cervical carcinogenesis. In the following clinical trial, 250 females infected with high-cancer-risk HPV16/18 (healthy and pre-cancerous) were recruited into a placebo-controlled clinical study of supplementation with fermented mangosteen (FM, 28g/day, daily) for three months. Our findings indicate that FM, and not a placebo, in combination with routine anti-viral therapy, could prevent, slow down, or even interrupt HPV-associated cervical carcinogenesis, mainly through the suppression of leukocyte recruitment into infected tissue, through anti-inflammatory effects, and through the restoration of nitric oxide metabolite-initiated TRAIL-dependent apoptosis.

**Abstract:**

In the observational clinical study, we identified the oxidative markers of HPV-associated cervical carcinogenesis and the local/circulating ligands of TNF-alpha-induced apoptosis. Cervical biopsies of 196 females infected with low-cancer-risk HPV10/13 or high-cancer-risk HPV16/18 (healthy, pre-cancerous CIN I and CIN II, and CIN III carcinoma) were analysed for OH radical scavenging, catalase, GSH-peroxidase, myeloperoxidase (MPO), nitrate/nitrite, nitrotyrosine, and isoprostane. Ligands of TNF-alpha-dependent apoptosis (TNF-alpha, TRAIL, IL-2, and sFAS) were determined in cervical fluid, biopsies, and serum. Cervical MPO was highly enhanced, while nitrotyrosine decreased in CIN III. Local/circulating TRAIL was remarkably decreased, and higher-than-control serum TNF-alpha and IL-2 levels were found in the CIN I and CIN III groups. Then, 250 females infected with HPV16/18 (healthy and with CIN I and CIN II) were recruited into a placebo-controlled clinical study of supplementation with fermented mangosteen (FM, 28g/day, daily) for three months. Post-trial colposcopy revealed normal patterns in 100% of the FM group versus 62% of the placebo group. Inflammatory cells in cervical fluid were found in 21% of the FM group versus 40% of the placebo group. Locally, FM drastically diminished MPO and NO_2_/NO_3_, while it remarkably increased TRAIL. Additionally, FM supplementation normalised serum TRAIL, TNF-alpha, and IL-2.

## 1. Introduction

Human papillomavirus (HPV) is connected with virtually all cases of cervical cancer. The viral infection-associated chronic inflammation has been considered as a leading risk factor for carcinogenesis in humans [1,2,3]. It has been estimated that 15–20% of cancers worldwide are attributable to virus, bacteria, or parasite infections causing chronic inflammation [4]. One of the characteristic features of chronic inflammation is the long-lasting overproduction of reactive oxygen and nitrogen species (ROS and RNS, respectively) released by inflammatory, epithelial, and endothelial cells [5,6,7]. From one side, ROS and RNS play multiple essential roles in the innate immune response to infectious agents [8,9], as well as in metabolism and the elimination of mediators of inflammation [10]. On the other hand, their excess could negatively affect the normal structure and functions of host cells and tissues, thus creating conditions for endogenous mutagen formation [11] and/or carcinogenic transformations [11,12,13]. For example, oxidative and nitration/nitrosylation metabolites that are formed upon the interaction of DNA with ROS and RNS are either mutagenic [11] factors causing tumour progression [14] or can induce carcinogenesis through the activation of proto-oncogenes and the inactivation of tumour suppressor genes [15,16,17].

Oxidative imbalance has been clearly recognised as a promoting factor in HPV-initiated carcinogenesis [18]. For example, while the expression of thioredoxin reductase 2 and glutathione-S-transferase peaked, inducible nitric oxide synthase (iNOS) was progressively reduced in dysplastic and neoplastic cervical tissue. Applying a redox proteomic approach, De Marco et al. came to the conclusion that the HPV16 neoplastic progression of cervical cancer is associated with the oxidative modifications (carbonylation) of DNA and proteins involved in cell morphogenesis and terminal differentiation in dysplastic tissues. In contrast, cancer tissues were characterised by the selective reduction of carbonyl adducts on detoxifying/pro-survival proteins that reflected an improved control of oxidative alterations [18].

The cytokines of the tumour necrosis factor (TNF) ligand family, such as the cell surface death receptor (sFAS), TNF, and TNF-related apoptosis-inducing ligand (TRAIL), are well-known regulators of apoptosis through their corresponding receptors [Ryu]. TRAIL was first identified as an inducer of p53-independent apoptosis in a variety of tumour cell lines and in cervical cancer patients (reviewed in [19]).

There are several evolving preventive strategies that could substantially reduce the burden from cervical carcinoma by the elimination of etiological factors and the inhibition of its development. First of all, they include HPV vaccines, diets enriched in fruits and vegetables [20,21,22,23], food supplements [23,24], and other chemopreventive agents [25,26,27,28,29,30]. Major molecular and cellular pathways for non-medicinal anti-cervical carcinoma preparations target the elimination/diminishing of the load of existing high-risk HPV infection [30,31,32], the restoration of normal non-viral microbiota [33] and redox balance [25,31,34,35,36,37,38], anti-inflammatory action [21,39], cell cycle influencers, and pro-apoptotic effects towards infected tumour transformed cells [40,41].

One of the most promising candidates, possessing several of the indicated activities, is fermented mangosteen (*Garcinia mangostana* Linn., Guttiferae), a popular botanical food supplement sold in large quantities around the world. Although well-known in ethnopharmacology since ages past, mangosteen-based products have been only recently studied for their phytochemical content and biological activities. Thus, the widely claimed cancer chemopreventive and anti-cancer effects of fermented mangosteen (FM) have been mainly attributed to the xanthons, tannins, and polyphenols of this health dietary additive [42,43,44,45]. These biological actives are mainly concentrated in the pod (synonyms: pericarps or rinds) [46] and seeds, non-edible parts of this delicious fruit usually referred to as “the queen of fruits”. The blue and purple colours of the rape mangosteen skin are associated with anthocyanines, resveratrol, quercetin, rutin, and ellargic acid, all of them known for numerous biological activities, including chemopreventive anti-cancer effects [26,27,28,40,41,47,48]. The white colour of the seed placenta, characteristic of the mangosteen pod, indicates the presence of beta-glucans and lignans with specific immunomodulating and anti-cancer actions, while the seed shells contain large amounts of lignins, which possess anti-cancer, anti-viral, and anti-microbial properties (reviewed in [49,50]). To increase the bioavailability of secondary plant metabolites encaged into the fibrous skin, mesopericarp, and seeds, they are subjected to a long-lasting controlled microbial and/or yeast fermentation.

Fermentation is the most ancient and the most natural way of plant food processing and preservation. During the fermentation (or external digestion) of plant parts, high molecular weight molecules, such as polysaccharides, glycolipids/glycoproteins, and nucleic acids of plant cell walls, seeds, peels, or pods, are decomposed to low molecular weight units (molecular moieties) that are readily absorbed through the gastrointestinal barrier and immediately available for a variety of metabolic processes [51]. Fermentation significantly enhances the cancer chemopreventive properties of non-fermented plants [52]. Moreover, fermented fruit preparations are enriched with the membrane fragments, lipoglycopeptide complexes, and exometabolites of fermenting yeasts and lactobacilli, probiotics that contribute to their immuno-modulating, nutritional, and physiological value [53,54]. Unfortunately, practically all research on the bioactivity of FM has been carried out in in vitro and ex vivo experiments, while clinical data are lacking.

The primary goal of the present study was the search for oxidative markers of HPV16- and HPV18-related cervical carcinogenesis and their possible connection with local and circulating ligands of TNF-alpha-induced apoptosis. In the first stage of the study, we found that the content of nitric oxide metabolites (nitrotyrosine and nitrates/nitrites) was significantly enhanced in the dysplastic cervical intraepithelial neoplasia (CIN) of different grades (CIN I and CIN II) groups, and the activity of MPO peaked in the invasive cervical cancer (CIN III) group.

Based on the results obtained, the placebo-controlled clinical trial on the clinical efficacy (the prevention and/or slowing down of carcinogenesis) of standardised FM gel, taken orally, was carried out. The secondary outcomes of the trial were oxidative and TNF-alpha-induced apoptosis ligand markers at systemic and cervical tissue levels in the practically healthy and the pre-cancerous CIN I and CIN II groups of female patients infected with HPV16/18, and in healthy females infected with low-risk HPV10/13.

## 2. Materials and Methods

### 2.1. Ethics Statement

All experiments with human material (blood, cervical biopsies, and cervical fluid) were carried out in accordance with the Helsinki Declaration, and the protocols of two clinical studies were reviewed and approved by the Ethical Committee of Berbekov’s Kabardino-Balkar State Medical University, Nal’chik, Russia (Protocol No. 133-2/2018 of 21st November 2018). The recruited patients and the healthy age-matched female donors signed an informed consent form.

### 2.2. Clinical Study Design and Recruitment Criteria

Two clinical studies were conducted. The first was an observational trial aimed at identifying local tissue oxidative markers of papillomavirus (HPV)-connected cervical carcinogenesis and their possible connection with local and circulating ligands of TNF-alpha-induced apoptosis. During the period from January 2019 to December 2019, 196 women attending the Gynaecology Department of Kabardino-Balkar Berbekov’s State University, Nal’chik, Russia, and presenting HPV infections of low (HPV10 and HPV13, n = 42; age range: 21-44 years) and high (HPV16 and HPV18, n = 154; for age range see Table 1) risk of cervical carcinogenesis, were recruited into the study after obtaining their informed consent. Fifteen age-matched females, not infected with HPV or other common viruses, such as cytomegalovirus (CMV) or herpes virus (HV1 and HV2), were invited to participate in the study as healthy controls. The demographic features, HPV infections, and clinical diagnoses of the participants in the first stage of the clinical observational study are collected in Table 1.

Women infected with HPV16 and HPV18 were diagnosed colposcopically and histologically on the basis of dysplastic lesions of different histological grades (n = 75; age range: 24–45 years; the pre-cancerous well-differentiated CIN I and moderately differentiated CIN II groups) or with clinically, histologically, and immunologically confirmed invasive cervical cancer (n = 45; age range: 28–53 years; the poorly differentiated CIN III group). The inclusion criteria were as follows: (a) patients older than 18 years; (b) not pregnant; (c) not infected with any other common viruses; (d) not bearing or have had any tumour apart from invasive cervical cancer in order to be included in the CIN III group; (e) not treated with any immune response-modulating or anti-viral drug for the last three months.

At the first visit, all participants were subjected to a full gynaecological examination. Cervical fluid containing ectopic cervical cells was collected and processed for cytological, immunological, and virological analyses. Biopsies were taken for standard histological evaluation, as well as for oxidative marker and apoptosis ligand assays. Of note, in the cases of dysplastic and neoplastic lesions, biopsies were taken from the area in a close vicinity (5 mm from the lesion border). All participants donated their venous blood for immunological analyses, as well. Any decision about the diagnostic procedures, treatment protocols, and follow-up period was based exclusively on the objective clinical picture, irrespective of any need of the observational trial.

Taking into account the outcome that several definite oxidative markers and TRAIL levels (in plasma and cervical fluid) corresponded to the severity of HPV-associated cervical dysplasia/neoplasia, we proceeded with a randomised, double-blind, placebo-controlled single-centre trial on the clinical efficacy and effects towards redox and apoptosis parameters of the FM food supplementation on HVP-infected patients with cervical dysplasia (CIN I and CIN II). A 5% honey solution in mineral water was used as the placebo. Females infected with HPV16 and HPV18 without clinical and colposcopic evidence of dysplasia (n = 152; age range: 25–45 years; practically healthy controls) and presenting clinical/colposcopic features of cervical dysplasia of different grades (CIN I or CIN II, n = 98; age range: 25–52 years) were invited to participate in the trial and signed informed consent forms. The inclusion criteria (a)–(c) and (e) were similar to those for the first observational clinical study. The criteria (d) were re-formulated as “not bearing or have had any tumour”. One more criterium was added: (f) no antioxidant and mineral supplementation for at least three months before entry to the trial. During the period from January 2019 to November 2019, two hundred and fifty women were enrolled and randomly placed into four groups: Group 1—practically healthy, placebo (n = 70); Group 2—practically healthy, FM supplementation (n = 82); Group 3—CIN I and CIN II, placebo (n = 48); Group 4—CIN I and CIN II, FM supplementation (n = 50). All patients with clinical symptoms of dysplasia received surgical removal of lesions. All the participants were prescribed conventional anti-viral therapy per os and in vaginal lavages. The patients’ demographic distribution and clinical data are summarised in Table 2.

In addition, patients from Groups 2 and 4 received FM supplementation as a standardised syrup (14 mL × 2 times a day at meal time for three months). The patients assigned to Groups 1 and 3 received 5% honey diluted in mineral water (14 mL × 2 times a day at mealtime for three months). FM was kindly provided free of charge by Carica Ltd., Manila, the Philippines.

As positive controls, the serum and cervical fluid of 30 healthy age-matching females without serum antibodies to HPV, herpes simplex types I and II, and cytomegalovirus (CMV), as well as free of HPV and CNV DNA in the cervical fluid, were used.

### 2.3. Food Supplement in Question

Standardised syrup of fermented mangosteen (FM) manufactured by Carica Ltd. (Manila, the Philippines), approved as a food supplement and distributed locally and abroad, was a kind gift of the manufacturer. The FM was produced from fruits collected from wild (non-cultivars and non-genetically modified) species of tropical *Garcinia mangostana* Linn. grown in remote, non-industrial parts of the Philippines. In brief, pods of rape fruit were separated from the edible flesh, washed, and mashed. The mash underwent the process of controlled fermentation by a food-quality culture of *Saccharomyces cerevisia* yeasts and *Lactobaccilus casei* within a 6-month period. Controlled enzymatic splitting was reached due to the strictly regulated temperature, oxygen, and nutrient supply, as well as frequent in-process quality tests. The fermentation process was stopped by adding the honey of wild bees (5%), followed by pasteurisation at 55 °C and filtration under pressure. The final FM product was a sour-sweet thick gel that was reddish-brown in colour. Post-fermentation analyses showed a high concentration of phenolics, fruit acids (pH 5.2), unsaturated fatty acids, sitosterols, macroelements, such as calcium, magnesium, and potassium, and microelements, such as copper, zinc, and iron. The ready FM product was free from pathogenic microorganisms and toxic elements, i.e., Sr, As, and Pb.

### 2.4. Clinical Diagnosis

All participants were subjected to a scrupulous gynaecological examination. The extended colposcopy with a magnification ×15 was carried out by a Letsegang colposcope (Germany) with video registration in order to identify and register the state of cervical epithelia. The cytological screening of cells that were collected from eczo- and endo-cervices by a spatula was performed by a conventional Papanicolaou smear test (Pap smear test).

### 2.5. Viral Analyses

Different types of HPV were determined by a type-specific PCR assay, using corresponding primers [55,56,57]. Total RNA was isolated using the GenElute Mammalian Total RNA kit (Sigma-Aldrich, Milan, Italy) and was reverse-transcribed using the iScript cDNA Synthesis kit (Bio-Rad, Hercules, CA, USA). cDNA was amplified with IQ SYBR green Supermix (Bio-Rad, Hercules, CA, USA), using the MiniOpticon Real-Time PCR Detection System (Bio-Rad, Hercules, CA, USA).

Herpes (HSV1 and HSV2) and cytomegalovirus (CMV) infections were assessed by real-time PCR, using the following primers: HSV1 fwd: CCT-TCG-AAC-AGC-TCC-TGG; rev: ATG-ACG-CCG-ATG-TAC-TTT-TTC-TT. HSV2 fwd: TCC-ATT-TTC-GTT-TTG-TGC-CGG; rev: ATG-ACG-CCG-ATG-TAC-TTT-TTC-TT. CMV fwd: ATG-ACG-CCG-ATG-TAC-TTT-TTC-T; rev: 5′-ACT-GGT-CAG-CCT-TGC-TTC-TAG-TCA-CC [Fax].

### 2.6. Biological Material Sampling and Processing

#### 2.6.1. Blood Sampling and Processing

Venous blood (6 mL) was taken into two hermetically sealed tubes and left for 1 h at room temperature for blood cell sedimentation. Serum was collected, aliquoted, and stored at −70 °C until the ELISA and PCR assays.

#### 2.6.2. Cervical Tissue Sampling and Processing

Biopsies were taken for standard histological pathological evaluation, as well as for oxidative marker and apoptosis ligand assays, in a colposcopy-guided manner. Of note, in the cases of dysplastic and neoplastic lesions, biopsies were taken from the area in a close vicinity (5 mm from the lesion border). The biopsies were placed in an ice-cold 0.1 M potassium phosphate buffer (pH 7.4) and thoroughly homogenised. The homogenates were centrifuged at 900× *g* and +4 °C for 40 min. The supernatants were collected and stored at −80 °C until they were analysed. The protein content in the supernatants was determined by Lowry’s method, which is described elsewhere.

#### 2.6.3. Cervical Fluid Collecting and Processing

Fluid containing ectopic cervical cells was collected from the walls of the cervical channel after its opening by a spatula, was centrifuged at 230× *g* for 10 min, and then the supernatant was stored for immunological analyses. The cervical cell sediment was washed twice with a potassium phosphate buffer and used for differential counting with an automatic Coulter counter (Beckman Coulter Inc., High Wycombe, UK) and for the HPV type-specific PCR assay.

### 2.7. Assays for Oxidative Markers

#### 2.7.1. Enzymatic Activities

The activities of pro- and antioxidant enzymes were measured by spectrophotometric methods: for MPO, the absorbance at 560 nm was recorded after the reaction with ortho-dianizidine; for catalase, the absorbance at 240 nm was recorded during the reaction with hydrogen peroxide; and for glutathione peroxidase, the absorbance at 412 nm was measured after the reaction with GSH in the presence of t-butyl peroxide. Homogenates were added to the reaction mixture at a concentration of 1 mg protein per mL, and the results were expressed in % of the control mixtures without homogenates.

#### 2.7.2. Hydroxyl Radical Scavenging

Hydroxyl radical scavenging capacity was assessed by luminol-dependent chemiluminescence in a Fenton reaction. In brief, the reaction was initiated by the addition of 17.7 μM FeSO_4_ to 0.01 M potassium phosphate buffer containing 17.7 μM hydrogen peroxide and 0.2 mM luminol. The integral square under the chemiluminescence spike was calculated and plotted against the value obtained in the absence of homogenate. The data were expressed in arbitrary units.

#### 2.7.3. Nitrotyrosine Determination

The levels of nitrotyrosine were measured in accordance with methods described previously [58,59]. The supernatants were assayed for 3-nitrotyrosine using a Nitrotyrosine ELISA kit (Northwest/AMS Biotechnology, Manchester, UK), following the manufacturer’s instructions. Briefly, the samples were incubated in the wells coated with a captured nitrotyrosine antibody and a biotinylated secondary tracer antibody. The addition of streptavidine-peroxidase, followed by tetra-methyl benzidine, resulted in colour development proportional to the nitrotyrosine levels, which were quantified spectrophotometrically at 450 nm. Total nitrate/nitrite levels were measured by Griess reaction using the analytical kit “Nitrate/nitrite Assay Kit Colorometric” (Sigma Co., Milan, Italy).

#### 2.7.4. Isoprostane Determination

The isoprostane content in the cervical tissue was determined as described in [60]. The homogenates were prepared on ice and the supernatants were diluted with distilled and de-ionised water. The solutions were acidified to below pH 4.0 and used for the further determination of free 15-F2t-IsoP by enzyme immunoassay (EIA) (Northwest NWLSSTM). Briefly, 15-F2t-IsoP in the samples or standards was allowed to compete with 15-isoprostane F2t conjugated to horseradish peroxidase (HRP) for binding to a polyclonal antibody specific for 15-isoprostane F2t coated on a micro plate. Subsequent tetra-methyl benzidine additions resulted in a blue colour development that was inversely proportional to the quantity of 15-isoprostane F2t in the original samples or standards. After the addition of a stop solution, the absorbance was read at 450 nm.

### 2.8. Assays for Ligands of Apoptosis

The ligands of TNF-dependent apoptosis were quantitatively determined in serum, circulating leukocytes, cervical fluid, and cervical wall cells. The protein levels of sFAS, TRAIL, TNF-alpha, and IL-2 were measured by the ELISA method, using corresponding kits and following the manufacturer’s instructions (BCM Diagnostics, Woodland, CA, USA).

Gene expression for sFAS and TRAIL in cervical biopsies was determined by quantitative real-time PCR, using TaqMan technology in accordance with the protocols of the European Anti-Cancer Program (EAC 2003). In brief, cervical tissue was soaked in an RNA-stabilising solution (RNA-later, Ambion, Austin, TX, USA) and stored at −20 °C for a period of not more than 3 months. The samples were homogenised on ice, and total RNA was isolated using an iPrepPureLink TM Total RNA kit (Invitrogen, Waltham, MA, USA). The primers for sFAS were: fwd: AAGCGGTTTACGAGTGACT; rev: TGGTTCCAGGTATCTGCTTC [Itoh]. Primers for TRAIL were: fwd: TGAAATCGAAAGTATGTTTGGGAATAGATG; rev: TGACGAAGAGAGTATGA ACAGCCCCTGCTG [61,62].

### 2.9. Statistics

Statistical analysis was performed using the STATISTICA 6.0 program from StatSoft Inc. The reported values were treated as continuous. The normality of the data was checked using the Shapiro–Wilk test. Since the distribution of the data was significantly different from normal, non-parametric statistics were used. The results were expressed as the mean ± SD. In some cases, values were presented as median, lower and upper quartiles, and minimum and maximum. The Mann–Whitney U test was employed for comparison between independent groups of data. To evaluate the difference between connected data, the two-tailed Student’s *t*-test was applied, and *p* values < 0.05 were considered to be significant.

Correlations between different oxidative markers, as well as between oxidative markers and ligands of TNF-alpha-induced apoptosis, were evaluated by the Pearson linear correlation coefficient. A *p* value < 0.05 was considered to be statistically significant. If necessary (comparison among three groups or more), *P*-values were adjusted for multiple comparisons, using the Bonferroni adjustment.

## 3. Results

### 3.1. Local Cervical Oxidative and Nitrosative Markers in HPV-Infected Female Patients at Different Stages of Cervical Carcinogenesis

**Figure 1:**
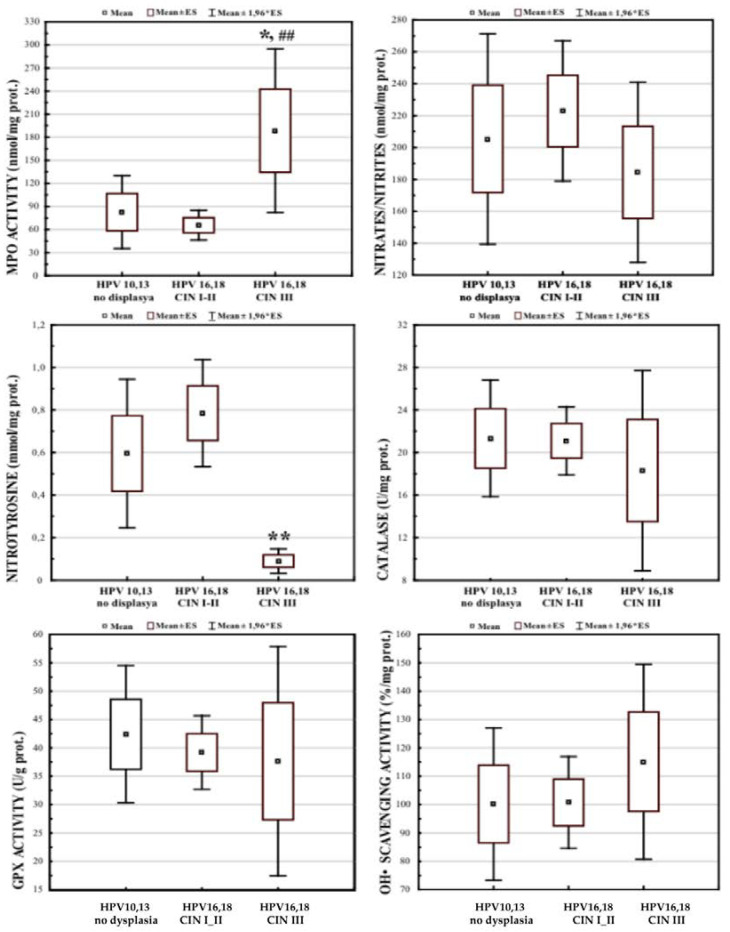
Markers of oxidative stress and antioxidant defence in cervical tissue of females infected with different types of human papillomavirus and at different stages of the disease. Sample size of groups: HPV10/13, n = 42; HPV16/18 CIN I and CIN II, n = 75; HPV16/18 CIN III, n = 45. * *p* < 0.05 versus HPV10/13 group; ## *p* < 0.05 versus HPV16/18 CIN I-II group; ** *p* < 0.01 versus HPV10/13 and HPV16/18 CIN I-II groups.

The measurement of selected markers of oxidative and nitrosative stress in the cervical biopsies (Figure 1) revealed a significant increase in MPO activity in the CIN III group as compared to the CIN I-II HPV16/18 group, and to the low oncogenic HPV10/13 non-dysplastic group. The levels of nitrotyrosine were significantly reduced in the CIN III group as compared to the CIN I-II and the HPV10 and HPV13 groups.

**Figure 2:**
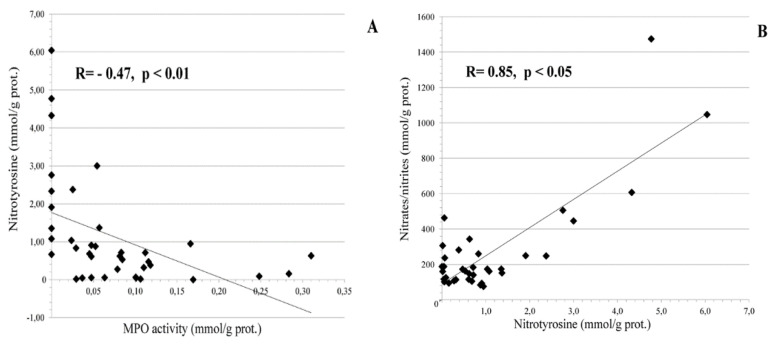
Correlations between redox parameters in cervical tissue of HPV-infected females with CIN I and CIN II (n = 75). (**A**) Correlation between MPO activity and nitrotyrosine content; (**B**) correlation between nitrotyrosine and nitrate/nitrite levels.

In the CIN I-II group, a strongly significant (*p* < 0.01) negative correlation between nitrotyrosine and MPO activity levels was found, as well as a positive significant (*p* < 0.05) correlation between nitrate/nitrite levels and nitrotyrosine, as shown in Figure 2.

### 3.2. Cervical and Circulating Ligands of TNF-Alpha Apoptosis in HPV-Infected Female Patients at Different Stages of Cervical Carcinogenesis

**Figure 3:**
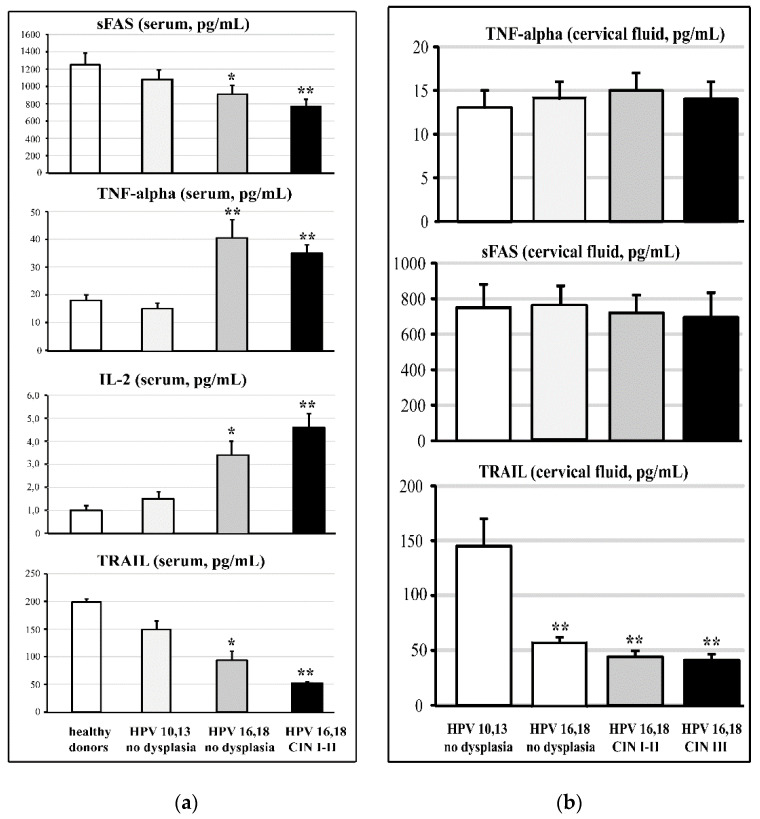
Serum (**a**) and cervical (**b**) ligands of TNF-alpha-induced apoptosis in females at different stages of HPV-associated carcinogenesis. Sample size of groups: HPV 10/13, n = 42; HPV 16/18 and healthy, n = 152; HPV 16/18 CIN I and CIN II, n = 75; healthy donors, n = 15. * *p* < 0.05 vs. healthy donors and low oncogenic HPV infection; ** *p* < 0.01 vs. healthy donors and low oncogenic HPV infection.

The data on TNF-alpha-induced apoptosis ligands in the serum and the cervical fluid are shown in Figure 3. The TRAIL levels were found significantly (*p* < 0.01) reduced in the cervical fluid of the HPV16 and HPV18 groups, both with CINI-II dysplasia and with CIN III cancerous lesions. Non-significant changes could be observed among the study groups for the TNF-alpha and sFAS ligands. The analysis of the serum levels of the circulating ligands of TNF-alpha-induced apoptosis revealed significant differences between the CIN I-II dysplastic group and both the healthy donors and the low oncogenic HPV-infected groups for sFAS, IL-2, and TRAIL (*p* < 0.05), and even more marked (*p* < 0.01) in the case of TNF-alpha. The difference versus healthy donors was highly significant (*p* < 0.01) for all ligands in the CIN III group.

**Figure 4:**
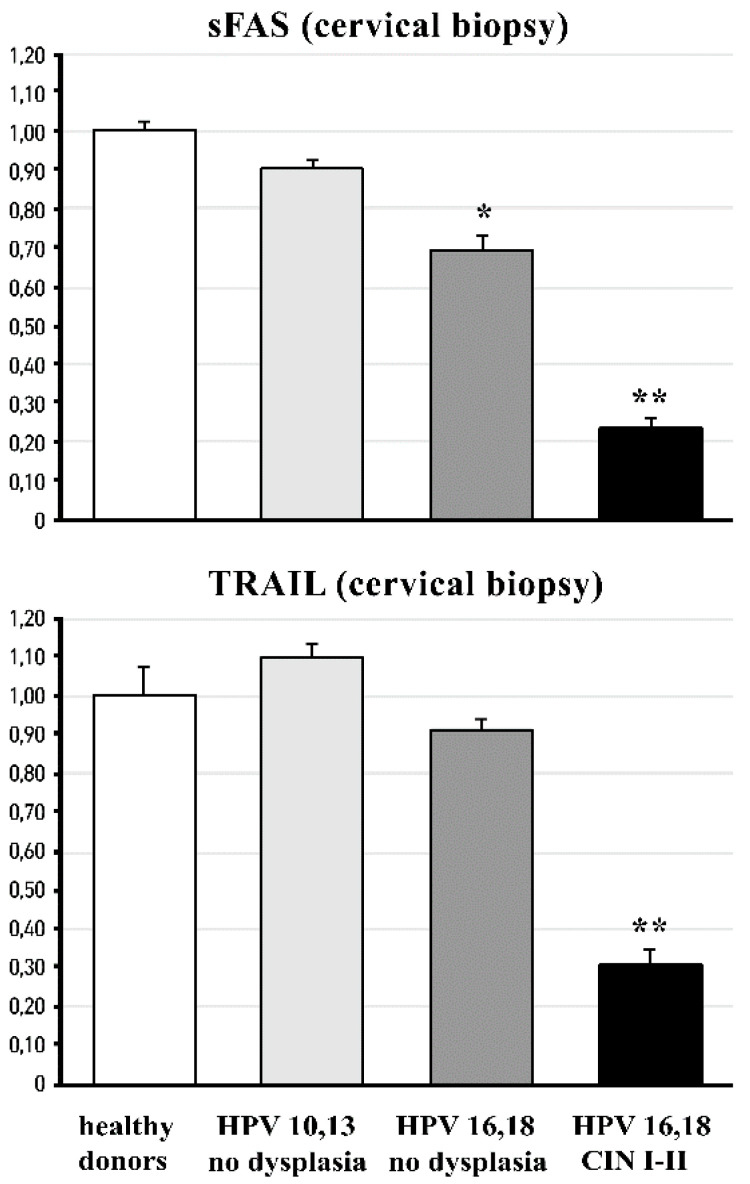
sFAS and TRAIL mRNA expression (arbitrary units) in cervical biopsies of females with HPV infection and of healthy donors. Sample size of groups: HPV 10/13, n = 42; HPV 16/18 and healthy, n = 152; HPV 16/18 CIN I and CIN II, n = 75; healthy donors, n = 15. * *p* < 0.05 ** *p* < 0.01 vs. healthy donors, and low and highly oncogenic HPV infection.

At the same time, the analysis of the mRNA expression of sFAS and TRAIL in cervical tissue (biopsies) showed a significant (*p* < 0.01) decrease in the expression of sFAS in the groups infected with high oncogenic HPV strains, both without dysplasia and with CIN I-II, as well as a marked (*p* < 0.01) reduction in TRAIL mRNA expression in the CIN I-II pre-cancerous group (Figure 4).

### 3.3. Effects of FM Supplementation on Macro-Histological Symptoms of Cervical Dysplasia

**Figure 5:**
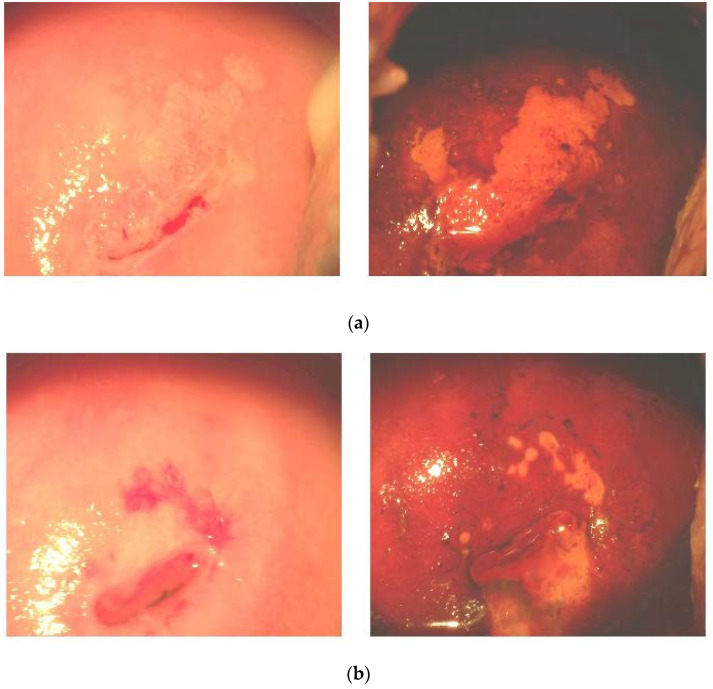
Typical colposcopy of CIN I and CIN II lesions before (**a**) and after (**b**) administration of antioxidants. (**a**) Before administration of antioxidants (ectopic transformed areas, squamous cell metaplasia, and mosaics marked by the iodine-negative areas). (**b**) After a three-month course of antioxidants (normal tissue basis, shrinkage of iodine-negative areas, and cervical coagulation). **Left** panels are colposcopic images without iodine staining. **Right** panels are colposcopic images after iodine staining.

The post-trial colposcopic examination revealed normal patterns in 100% of the FM group versus 62% of the placebo group. A sample of macro-histological effects of FM supplementation is shown on Figure 5, where the (a) panel photos were taken in the beginning of the trial, and the panel (b) photos were taken at the cessation of it. Before the administration of FM, ectopic transformed areas, squamous cell metaplasia, and mosaics marked by the iodine-negative areas were evident. After a three-month course of FM, we observed normal tissue basis, the shrinkage of iodine-negative areas, and cervical coagulation. Inflammatory cells in cervical fluid were found in 21% of the FM group versus 40% of the placebo group.

### 3.4. Effects of FM Supplementation on Oxidative and Nitrosative Markers in Cervical Tissue

The cervical tissue markers of oxidative and nitrosative stress appeared to be selectively modified by the FM treatment of the CIN I-II patients (Table 3). After 3 months, MPO activity was significantly (*p* < 0.01) decreased, as well as nitrate/nitrite and nitrotyrosine levels (*p* < 0.05). Catalase activity showed a trend toward reduction, whilst isoprostane and GSH-peroxidase differences, before and after FM treatment, were not detected at the cervical level.

### 3.5. Effects of Fermented Mangosteen Supplementation on Systemic and Topical (Cervical) Ligands of TNF-Alpha Apoptosis

**Figure 6:**
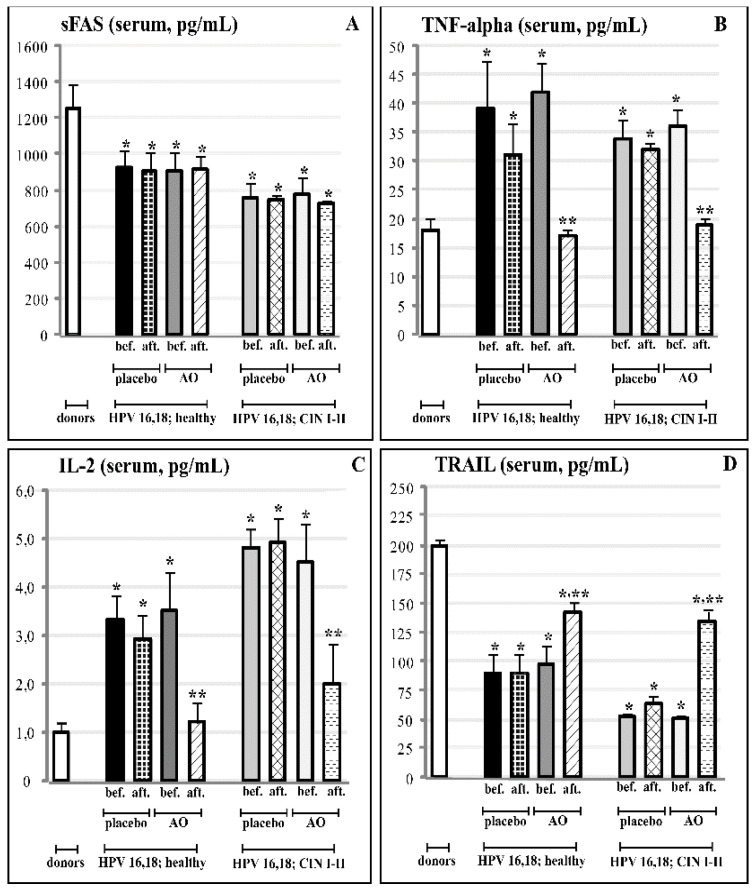
Effects of FM supplementation (AO) or placebo on serum content of the apoptosis ligands (**A**–sFAS; **B**–TNF-alpha; **C**–IL-2; **D**–TRAIL) in groups of practically healthy HPV16- and HPV18-infected females (n = 152), and in groups of HPV16- and HPV18-infected females with pre-cancerous cervical lesions (CIN I and CIN II) (n = 98). * *p* < 0.05 vs. donors; ** *p* < 0.05 vs. before treatment.

**Figure 7:**
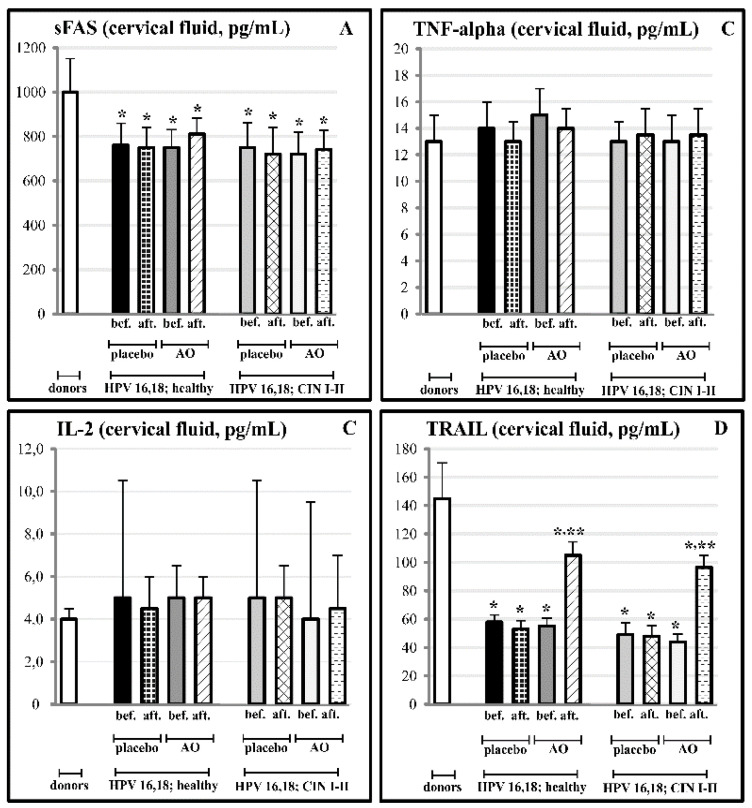
Effects of fermented mangosteen (FM) supplementation on cervical fluid content of the apoptosis ligands (**A**–sFAS; **B**–TNF-alpha; **C**–IL-2; **D**–TRAIL) in groups of practically healthy HPV16- and HPV18-infected females (n = 152) and in groups of HPV16- and HPV18-infected females with pre-cancerous cervical lesions (CIN I and CIN II) (n = 98). * *p* < 0.05 vs. donors; ** *p* < 0.05 vs. before trial.

The results of the three-month-long course of FM supplementation are collected in Figure 6 and Figure 7. The FM treatment remarkably affected circulating ligands of TNF-alpha-induced apoptosis in both experimental groups with HPV16- and HPV18-infected females, with or without clinical features of cervical carcinogenesis. In these groups, highly enhanced serum levels of TNF-alpha and IL-2 went down to normal values (Figure 6B,C).

At the same time, strongly suppressed serum content of TRAIL was statistically significantly restored, although TRAIL levels did not reach the normal values (Figure 6D). The FM supplementation did not induce any significant change in circulating sFAS ligands; we also did not observe any effects on circulating ligands in both placebo groups (Figure 6A).

Regarding the cervical levels of the same ligands, the course of FM, and not of the placebo, led to the statistically significant enhancement of TRAIL, while, again, it did not affect suppressed levels of sFAS (Figure 7A,D). At the cervical level, the background concentrations of TNF-alpha and IL-2 did not differ in HPV-infected females with and without pre-cancerous lesions. They remained within the background range after the cessation of the clinical trial with FM (Figure 7B,C).

## 4. Discussion

Clinical and epidemiological studies have revealed that certain pathogens causing persistent infections and chronic inflammation were associated with cancer [1,9,63]. The oncogenic action seems to be induced, maintained, and mediated by chronic inflammation and/or by alterations in host cells caused by the microbial genome [9,64]. Here, we observed (Figure 1) two molecular markers of chronic inflammation, MPO and nitrotyrosine, which were significantly changed in the cervical fluid of the females with cervical cancer (CIN III). As expected, there was a strong positive correlation between NO_2_^−^/NO_3_^−^ and nitrotyrosine, the latter being formed under nitration of the amino acid by these nitrative species (Figure 2A). At the same time, we found a quite significant negative correlation between MPO and nitrotyrosine (Figure 2B). Human granulocytes release MPO from intra-cytoplasmic granules being challenged by microbes or viruses [17]. At the same time, they contain very low levels of inducible nitric oxide synthase [14]. Hence, they do not generate reactive nitrogen species intracellularly [65]. Instead, human granulocytes are likely to use nitrite (NO_2_^−^) from other sources, thus forming nitrating oxidants and, as a consequence, nitrotyrosine in the extracellular environment [13]. Nitrating oxidants are implicated in the host defence and pathogenesis of many diseases [13,15,16]. Nitrative stress contributes to asbestos-induced carcinogenesis [10] through myeloperoxidase (MPO), which plays a crucial role in the asbestos-derived inflammatory response and following asbestos-induced carcinogenesis [11,12,13]. In the cystic fibrosis airways, MPO acts as a phagocyte-derived NO oxidase that diminishes NO bioavailability and, consequently, its immune protective (anti-bacterial and anti-viral) and anti-inflammatory properties [66]. On these grounds, it has been suggested that MPO could be a target for therapeutic intervention to attenuate the oxidative burden and preserve essential physiological functions of NO. The inflammatory environment in HPV-infected cervical tissue provides all the factors necessary for the generation of nitrating agents by MPO, granulocyte invasion, and a high local level of NO_2_^−^ [13,15,16].

Irreversible tyrosine modifications by inflammatory oxidants such as peroxynitrite include the formation of characteristic markers 3-nitrotyrosine and 3,3′-dityrosine [67,68]. These modifications of critical tyrosine residues in proteins inactivate a variety of enzymes [67] and affect their structures [69]. Of great importance, tyrosine nitration may interfere with signal transduction pathways involving both the kinase-dependent and auto-phosphorylation of tyrosine. Thus, in epidermoid carcinoma cells, tyrosine nitration altered the epidermal growth factor receptor (EGFR) activation through irreversible dimerisation [70]. An alteration of EGFR-mediated signalling pathways in epithelial cells would inevitably affect cell proliferation or differentiation, which may bear implications on carcinogenesis [71,72,73].

Solid tumours (human gastric, colorectal, cervical, and bronchoalveolar carcinomas, as well as epithelial ovarian cancer) frequently contain inflammatory cells, such as neutrophils, macrophages, and T-lymphocytes [74,75,76,77]. Their recruitment is mainly explained by C-X-C chemokines, namely IL-8 (a potent chemoattractant for neutrophils) or C-C chemokines (MCP-1) [63]. The inflammatory cells recruited to tumours become an important additional source of the chemokines [14]; thus, a vicious cycle is formed. It is well-established that activated neutrophils induce prolonged DNA damage in neighbouring cells [78], mainly through the genotoxic effects of ROS and RNS [79,80]. These species are widely recognised as key carcinogenic agents, causing tumour transformation, progression, and metastasis.

As per comprehensive review [81], HPV-encoded intracellular proteins can reshape signalling pathways in a mode that facilitates carcinogenesis through escaping immune surveillance and the impairment of TRAIL-mediated apoptosis. In the present study, we found that circulating levels of two cytokines (TNF-alpha and IL-2) and two ligands (sFAS and TRAIL) contributing to TRAIL-mediated apoptosis were significantly compromised versus normal values in females infected by highly oncogenic HPV forms without any symptoms of cervical pre-cancerous lesions as yet (Figure 3A). The impairment was aggravated in females with pre-cancerous CIN I and CIN II stages, while no differences were seen between healthy non-infected HPV females and those infected by low-risk HPV 10,13. When TNF-alpha, sFAS, and TRAIL proteins were measured in the cervical fluid, there were no differences between groups in the content of sFAS and TNF-alpha; however, local levels of TRAIL were dramatically diminished in all HPV16/18-infected groups of females (with no lesions, CIN I-II, and CIN-III stages) (Figure 3B). At the level of mRNA for sFAS and TRAIL in cervical tissue, the remarkable suppression of both was observed in the group with pre-cancerous states (CIN I-II). In the groups with no dysplasia, infected either by low- or high-risk HPV, mRNA expression was practically at normal levels.

A new approach to target and kill circulating tumour cells has been proposed recently [82]. The method is based on the coating of circulating leukocytes with liposomes loaded by TRAIL. The coated leukocytes could be considered as “un-natural killer cells”, because they resemble natural killers activated by IL-2, which overexpresses TRAIL to attack and induce apoptosis in cancer cells. Applying the immunohistochemical approach, Carrero and co-authors [77] showed that in the advanced stage of pre-malignant lesions of the cervix, a progressive stage-dependent CD3/VEGF-positive lymphocyte infiltration correlated with the increased number of superoxide-producing cells, while tissue levels of nitrites and nitrates remained unchanged. However, in this work, only 16% of patients with CIN were infected with HPV; hence, one cannot estimate the impact of oxidative stress on virus-associated carcinogenesis.

Nutritional paradigms and dietary active components in the chemoprevention of carcinogenesis and in the potentiation of anti-cancer therapies have drawn a lot of attention recently (reviewed in [9,20,21,22,23,24,25]). Resveratrol has been shown to potentiate the apoptotic effects of TRAIL and other death cytokines, as well as chemotherapeutic agents and gamma irradiation [47,83,84]. On these grounds, a novel strategy to enhance the efficacy of TRAIL-targeting chemoprevention/therapies has been suggested. However, recent publications raised serious concerns about the feasibility of resveratrol in anti-cancer therapy and possibly in cancer chemoprevention due to its poor pharmacokinetics, low potency, and nephrotoxicity [84]. The publication of our group [85] clearly showed the strong photo-toxicity of resveratrol, which is widely promoted for the prevention of UV-induced skin tumours. The anti-proliferative effect of the dietary anti-carcinogenic compound phenyl ethyl isothiocyanate in human cancer stem cells, derived from the human cervical tumour-derived HeLa cell line, partially resulted from the up-regulation of death receptors DR4 and DR5 of the TRAIL-mediated apoptotic pathway [86]. The dietary substances cucurbitacins, glycosylated triterpenes [87], luteolin [88], and epigallocatechin gallate [89] have been shown to regulate DNA repair systems and TRAIL-driven signalling in a number of cancer cells.

FM has been suggested as an effective chemopreventive/anti-cancer dietary product on the basis of phytochemical analyses [42], the in vitro and in vivo experiments [40,43,44,45] showing that its active components, such as polyphenols, terpenes, and xanthons, acted by different mechanisms, i.e., mTOR, deranged cell cycle, autophagy, and p53-dependent apoptosis. Direct antioxidant effects have been shown, as well [44,45]. Clinical data on the anti-cancer or cancer chemopreventive efficacy are completely lacking. In the present placebo-controlled clinical study, we showed, for the first time, that a 3-month-long supplementation with FM in females infected by HPV16/18 of high oncogenic risk, with the first symptoms of cancerous dysplasia (CIN I-II), resulted in the normalisation of macro-histological patterns in cervical tissue (100% of patients) and diminished the invasion of inflammatory neutrophils into the cervix (Figure 5). Along with the observed clinical efficacy, FM supplementation significantly suppressed the MPO presence, nitrative agents NO_2_^−^/NO_3_^−^, and nitrotyrosine in cervical tissue (Table 3). All these species are known for their carcinogenic action (see above). The most impressive findings were on the effects of FM supplementation on the TRAIL protein, circulating as well as localised, in the cervical fluid (Figure 6 and Figure 7). This TNF-alpha-related apoptosis-inducing ligand, an inducer of cancer cell apoptosis through death receptors [84], was initially greatly suppressed in HPV16/18-infected females without symptoms of dysplasia; gradually, its suppression became deeper in HPV16/18-induced cervical dysplasia (CIN I and CIN II) and cervical cancer (CIN III) (Figure 4). Supplementation with FM, and not with the placebo, led to the significant restoration of circulating and local TRAIL levels. Unusually low TRAIL levels in the cervical fluid in the beginning of the study were not accompanied by any significant change in the three other cytokines and ligands (TNF-alpha, IL-2, and sFAS), while the circulating TNF-alpha and IL-2 in serum were highly increased in all HPV16/18-infected females with or without signs of pre-cancer. That, in our opinion, could reflect the generalised chronic inflammatory response to viral infection. Of great importance, FM supplementation completely normalised the circulating levels of both pro-inflammatory cytokines, thus diminishing the burden from chronic inflammation.

On the grounds of the results described here and the literature data, we assumed that FM could exert preventive effects on HPV-induced cervical cancer through several pathways, where one or another of its numerous active ingredients could:

- theoretically diminish the load of high-risk HPV [28];
- regulate neutrophil chemotaxis and the degranulation of MPO-containing granules at sites where the virus is harboured;
- increase the bioavailability of NO to fight viruses that would also lead to diminished levels of potentially carcinogenic nitrative agents and nitrotyrosine;
- down-regulate the overexpression of inflammatory cytokines involved in chronic general inflammation;
- deeply affect TRAIL-related events, such as the apoptosis of HPV-infected cells, preventing them from cancerous transformation;
- the presence of numerous active compounds derived from the plant and fermenting microbes in the natural matrix of FM could attenuate their potential individual toxicity, increase bioavailability, and exhibit a synergy between them.

## 5. Conclusions

Our findings indicate that FM, in combination with anti-viral therapy, could prevent or slow down HPV-associated cervical carcinogenesis, mainly through the suppression of leukocyte recruitment into infected tissue, suppression of general virus-associated chronic inflammation, and restoration of nitric oxide metabolite-initiated TRAIL-dependent apoptosis.

Much more research is needed to elucidate the mechanism(s) of FM’s cancer-preventive effects. There are still many critical issues in the wide acceptance of fermented fruits as remedies for health problems, such as the lack of reliable clinical evidence, as well as scarce data on individual active components in fermented fruits and on the molecular mechanisms of their cancer chemopreventive effects. There is also an urgent need to optimise/standardise technologies of fermentation and to develop drugs based on the actives of fermented products.

## Figures and Tables

**Table 1:** Demographic and clinical data of women—participants in the observational study on the oxidative markers and TNF-alpha apoptosis ligands in HPV infections associated with cervical carcinogenesis.

Group	Number of Subjects	Age Range
HPV10, HPV13, healthy	42	21–44 year
HPV16, HPV18, CIN I + CIN II	75	24–45 year
HPV16, HPV18, CIN III	45	28–53 year
Healthy female controls	15	25–47 year

**Table 2:** Demographic and clinical data of women—participants in placebo-controlled trial on the clinical efficacy, oxidative markers, and TNF-alpha ligand levels of fermented mangosteen (FM) supplementation.

Group	Number of Subjects	Age Range
HPV16, HPV18, no dysplasia **Group 1**—placebo **Group 2**—fermented mangosteen (FM)	152 70 82	25–45 year
HPV16, HPV18, CIN I + CIN II **Group 3**—placebo **Group 4**—fermented mangosteen (FM)	98 48 50	25–52 year
Healthy controls without viral infections	30	27–42 year

**Table 3:** Oxidative markers in cervical tissue of CIN I and CIN II patients (n = 50) treated with fermented mangosteen for 3 months. * *p* < 0.05; ** *p* < 0.01.

Marker	Before Treatment	After Treatment
Myeloperoxidase, nmol/mg	76 ± 12	5.9 ± 1.3 **
Nitrates/Nitrites, nmol/mg	207 ± 18	147 ± 30 *
Catalase, U/mg	20 ± 1.8	15 ± 3
Isoprostanes, ng/mg	1.1 ± 0.2	0.9 ± 0.2
Nitrotyrosine, nmol/mg	1.1 ± 0.2	0.5 ± 0.3 *

## Data Availability

The data can be shared upon request.
